# Expression Profile of Circular RNAs in Oral Squamous Cell Carcinoma

**DOI:** 10.3389/fonc.2020.533616

**Published:** 2020-11-27

**Authors:** Yanxiong Shao, Yuhan Song, Siming Xu, Siyi Li, Haiwen Zhou

**Affiliations:** ^1^Department of Oral Mucosal Diseases, Shanghai Ninth People’s Hospital, College of Stomatology, Shanghai Jiao Tong University School of Medicine, Shanghai, China; ^2^National Clinical Research Center for Oral Diseases, Shanghai, China; ^3^Shanghai Key Laboratory of Stomatology & Shanghai Research Institute of Stomatology, Shanghai, China; ^4^Department of Oral Maxillofacial-Head and Neck Oncology, Shanghai Ninth People’s Hospital, College of Stomatology, Shanghai Jiao Tong University School of Medicine, Shanghai, China

**Keywords:** oral squamous cell carcinoma, circRNA, pathogenesis, non-coding RNA, sequencing

## Abstract

**Background:**

Circular RNAs (circRNAs) are involved in the pathogenesis of several diseases. Among oral maxillofacial cancers, oral squamous cell carcinoma (OSCC) has the highest incidence. However, the role of circRNAs in OSCC is still not clear. The aim of our study was to evaluate the circRNA expression profile in OSCC and explore further the potential role of circRNAs in the pathogenesis of OSCC.

**Methods:**

CircRNA sequencing was performed in 6 pairs of samples of OSCC and normal oral mucosal tissues. Expression of selected circRNAs was validated by qRT-PCR. GO and KEGG analyses were performed and binding relationships between circRNAs and miRNAs were predicted. The hsa_circ_0001766/miR-877-3p/VEGFA axis was selected to further elucidate its role in OSCC.

**Results:**

We showed that there were 122 differentially expressed (DE) circRNAs. Eight out of 10 selected circRNAs were validated by qRT-PCR. GO and KEGG analyses indicated that the identified DE circRNAs might be involved in the progression of OSCC. Then, after identification by Sanger sequencing and RNase R assay, the upregulated hsa_circ_0001766 was selected to investigate its potential role in OSCC. Bioinformatics analysis showed that hsa_circ_0001766 might act as a competing endogenous RNA (ceRNA) that sponged miR-877-3p to upregulate VEGFA expression. We selected OSCC cell lines SCC9 and SCC25. PCR results showed that the expression of hsa_circ_0001766 and VEGFA was upregulated in SCC9 and SCC25. Subsequently, using western blot, PCR, CCK8, and colony formation assays, we found that knocking down circRNA0001766 inhibited the expression of VEGFA and the proliferation of OSCC cells. Following this, miR-877-3p inhibitor reversed the inhibitory effect of si-hsa_circ_0001766 on expression of VEGFA and proliferation of OSCC cells.

**Conclusions:**

In conclusion, our study revealed the possible role of circRNAs in the pathogenesis of OSCC, and identified the potential role of the hsa_circ_0001766/miR-877-3p/VEGFA axis in OSCC progression.

## Introduction

Oral squamous cell carcinoma (OSCC) is the commonest cancer in the oral maxillofacial region, and is often accompanied by regional lymph node metastasis and even distant metastasis. However, the pathogenesis of OSCC is still unclear and there are no effective or satisfactory treatments available currently. According to reports, the 3-year overall survival rates for low-risk, intermediate-risk, and high-risk OSCC are 93, 70.8, and 46.2%, respectively, and the current 5-year survival rate for OSCC is 63% (1). Molecularly-targeted anti-tumor drugs have been widely used in various cancers clinically in recent years, since they have less side effects and better efficacy. Therefore, looking for key molecules in the pathogenesis of OSCC is of vital importance in the treatment for OSCC.

Circular RNAs (circRNAs) are a novel class of non-coding RNAs that are mainly distributed in eukaryotic cells and are over hundreds of nucleotides in length. CircRNAs are characterized by covalent closed loop structures with neither 5′ or 3′ polarities nor polyadenylated tails, which renders them resistant to degradation by RNA exonuclease and endows them with high conservatism and stability (2). The circRNAs discovered so far are mainly structured by head-to-tail splicing of exons (3). There are other types of circRNAs, such as intronic and sense overlapping ones.

Produced by back-splicing, most circRNAs distribute in the eukaryotic cells’ cytoplasm and have tissue and disease specificity. CircRNAs play an important role in the development of various diseases through regulation of gene expression by processes, such as transcription, translation, and splicing. In addition, numerous microRNA (miRNA) binding sites are located on circRNAs, which makes circRNAs the competing endogenous RNA (ceRNA) of miRNAs (4). Functioning as a miRNA sponge, circRNA can ameliorate the inhibitory effect of miRNA on its host gene and promote gene expression.

Stomatology researchers have expressed concern about circRNAs’ role in the pathogenesis of multiple diseases, such as oral squamous cell carcinoma (5–10), oral mucosal melanoma (11), and mucoepidermoid carcinoma of salivary glands (12). Regarding OSCC, Chen et al. (5) found that circRNA_100290 could regulate the function of cyclin-dependent kinase 6 (CDK6) by miR-26 sponging. Wang et al. (6) speculated that circDOCK1 is related to cellular apoptosis in OSCC. The knockdown of circDOCK1 expression and the upregulation of miR-196a-5p expression led to a decrease in BIRC3 formation and an increase in apoptosis in OSCC cells. Qiu et al. (7) detected 322 differentially expressed (DE) circRNAs between human tongue squamous cell carcinoma and corresponding adjacent tissues through high-throughput sequencing. Wang et al. (8) identified that circ_000334, circ_006740, and circ_006371 were significantly downregulated in OSCC and could act as ceRNA participating in the development of OSCC. Ouyang et al. (9) demonstrated that knockdown of has_circ_0109291 inhibited proliferation and migration of OSCC cell lines *in vitro*. Su et al. (10) verified that has_circ_0007059 was downregulated in OSCC and suppressed cell growth, migration, and invasion of SCC15 and CAL27 cells *via* AKT/mTOR signaling. Despite the putative role of circRNAs in OSCC reported in previous studies, the results of circRNA sequencing in OSCC varied in different studies, perhaps because of the limited amounts of samples (due to high costs), different control group selection, varied sites of sampling, and significant individual differences.

In this study, we aimed to evaluate the circRNA expression profile in OSCC and normal oral mucosa tissue samples and further explore the ceRNA networks and the potential role of circRNAs in the OSCC.

## Materials and Methods

### Patients and Samples

Twelve patients were enrolled in this research at the Shanghai Ninth People’s Hospital from August 2018 through December 2018. OSCC tissues were obtained from 4 males and 2 females (n = 6) with a histopathological diagnosis of OSCC and subjected to primary surgical treatment in the Department of Oral and Maxillofacial-Head and Neck Oncology. Normal oral mucosal tissues were obtained from 2 males and 4 females (n = 6) who underwent plastic surgery in the Department of Plastic and Reconstructive Surgery. All tissue samples were frozen in liquid nitrogen immediately after resection and stored at −80°C until RNA extraction. Patients with a previous history of chemo- or radiotherapy, and those suffering from severe diseases, including but not limited to cardiac dysfunction, hepatic and renal dysfunction, as well as pregnant women were excluded. Written informed consent was taken from all subjects prior to sample collection. The Ethics Committee of Shanghai Ninth People’s Hospital approved this study.

### RNA Extraction

Trizol reagent (Life Technologies, Carlsbad, CA, USA) was used to extract total RNA from tissue samples. The concentration and purity were detected by the NanoDrop ND1000 (Thermo Fisher Scientific, Waltham, MA, USA). All RNA samples passed the quality control according to the OD260/OD280 readings (1.8–2.1).

### CircRNA Sequencing

CloudSeq Biotech (Shanghai, China) provided high-throughput sequencing service. CircRNA high-throughput sequencing and bioinformatics analysis were done by Cloud-Seq Biotech. After ribosomal RNAs (rRNAs) were removed from the total RNA using Ribo-Zero rRNA Removal Kits (Illumina, USA), RNA libraries were constructed by using rRNA-depleted RNAs with TruSeq Stranded Total RNA Library Prep Kit (Illumina). The quality of libraries was controlled by the BioAnalyzer 2100 system (Agilent Technologies, USA). Libraries of 10 pM were denatured as single-stranded DNA molecules and sequenced for 150 cycles on Illumina HiSeq 4000 Sequencer (Illumina, San Diego, CA, USA).

### CircRNA Profiling Analysis

Paired-end reads were harvested by Illumina HiSeq 4000 sequencer, and the quality was controlled by Q30. After 3′ adaptor-trimming and removal of low-quality reads by cutadapt software (v1.9.3), the high-quality trimmed reads were left for circRNA analysis. Then, the high-quality reads were aligned to the reference genome/transcriptome with STAR software (v2.5.1b). CircRNAs were detected and identified by DCC software (v0.4.4). edgeR software (v3.16.5) was used to normalize the data and analyze DE circRNAs. CircRNAs with a fold change (FC) in expression ≥ 2.0 (*P* < 0.05) between the OSCC and the control samples were considered statistically significant and selected for further analysis. GO and KEGG analyses were performed. Binding relationships between circRNAs and miRNAs were predicted by TargetScan 7.0 and miRanda v3.3a. The network of circRNAs, miRNAs, and mRNAs was constructed with Cytoscape 3.1 software.

### Quantitative Real-Time Polymerase Chain Reaction (qRT-PCR) Validation

qRT-PCR was used to identify the selected circRNAs in the circRNA profile. Total RNA (2 μg), dNTPs Mix (HyTestLtd, Turku, Finland), SuperScript III Reverse Transcriptase (Thermo Fisher Scientific, Chino, CA, USA), and RNase Inhibitor (Enzymatics, GreenBay, Wisconsin, USA) were used for cDNA synthesis. GAPDH served as an endogenous control gene for circRNAs. qRT-PCR was performed by QuantStudio 5 Real-Time PCR System (Thermo Fisher Scientific). The experiment was duplicated in three times. Data analysis was performed by the 2-△△Ct method.

The primers were designed as follows: hsa_circ_0000857, 5’-CAGACGGACCCCACAGAC-3’ (forward) and 5’-TGGCGTTGAAACTGGGAT-3’ (reverse); hsa_circ_0006151, 5’-GGCACCAGCATAGCCAGT-3’ (forward) and 5’-CCAGGCGCTTTTCACAGT-3’ (reverse); hsa_circ_0005050, 5’-CCCAGCTGCTAGAGAACCA-3’ (forward) and 5’-TTGGTTGCCTGCTGGATT-3’ (reverse); hsa_circ_0001766, 5’-CCCATTCCTGTTGCCAAG-3’ (forward) and 5’-TCCTCCTCCTCCTCCTCTTC-3’ (reverse); hsa_circ_0032822, 5’-AGAAAGGCAGGAGCAGCTT-3’ (forward) and 5’-TCCAGCTGACCACGATGA-3’ (reverse); hsa_circ_0001470, 5’-TGCTCCAACAGGGGATGT-3’ (forward) and 5’-CGTCTCATTCCACAAGCCT-3’ (reverse); hsa_circ_0004390, 5’-CGGAGGACACCCATGAAG-3’ (forward) and 5’-GGCAGAAAAACGTCCCAA-3’ (reverse); hsa_circ_0104368, 5’-ATGGCGAATGTGGCTGAT-3’ (forward) and 5’-TAGTTGACCCGAGCTGCC-3’ (reverse); hsa_circSENP2, 5’-CCTCAACAGCTGAATGGGA-3’ (forward) and 5’-CCCACATCTCCCCCTTCT-3’ (reverse); hsa_circ_0008603, 5’-TCAAAGGTGGATTGGGGA-3’ (forward) and 5’-CATCCAGCATGGTGAGCA-3’ (reverse); VEGFA, 5’- AGGGCAGAATCATCACGAAGT-3’ (forward) and 5’- AGGGTCTCGATTGGATGGCA-3’ (reverse). miR-877-3p, 5’- TCCTCTTCTCCCTCCTCCCAG-3’. GAPDH, 5’-GGCCTCCAAGGAGTAAGACC-3’ (forward) and 5’-AGGGGAGATTCAGTGTGGTG-3’ (reverse). U6, 5’- CTCGCTTCGGCAGCACA-3’ (forward) and 5’- AACGCTTCACGAATTTGCGT-3’ (reverse)

### RNase R Assay and Sanger Sequencing

After total RNAs were extracted, RNA solutions were treated with RNase R (20U/μl, Epicentre) and the treated RNAs were detected by qRT-PCR. RNase R could digest linear RNA molecules, but not circRNAs. So, the change of results of PCR could determine the circular structure of RNAs. Additionally, Sanger sequencing was performed to verify the sequence and head-to-tail splicing structure of circRNAs.

### RNA Interference

Small interfering RNA (si-RNA) targeting hsa_circ_0001766 (5’GATTCTTCTAACA3’) and an inhibitor targeting miR-877-3p (5’GGGAGGAGGGAG3’) were constructed (Shanghai Asia-Vector Biotechnology Co., Ltd). Non-targeting control siRNA and inhibitor negative control were used as controls. Cells were transfected using Lipofectamine 3000 transfection reagent (Invitrogen, USA).

### Cell Culture

OSCC cell lines SCC9 and SCC25 were obtained from Cell Bank of China. Cells were cultured in DMEM/F12 culture medium supplemented with 10% fetal bovine serum (FBS). HOK cells were purchased from BeNa Culture Collection and were grown in RPMI 1640 medium with 10% FBS. All cells were incubated at 37°C in a humidified atmosphere of 5% CO_2_.

### Western Blotting

SCC9 and SCC25 cells were collected and total protein was collected using RIPA lysis buffer. The following antibodies and reagents were used: anti-VEGFA (Abcam, ab46154), anti-GAPDH (Cell Signaling Technology, #5174), and anti-rabbit IgG (Cell Signaling Technology, #7074). Then, samples were subjected to western blot.

### Cell Viability and Colony Formation Assays

Cell viability was tested using Cell Counting Kit-8 (CCK-8, Dojindo) assay according to the manufacturer’s guidance. SCC9 and SCC25 cells were incubated in an incubator at 37°C for 0, 24, 48, 72, and 96 h. The cell viability was determined by the absorbance at 450 nm using a microplate reader (Bioteck, Epoch2). About 1,000 cells/well were plated in six-well plates. Cells were incubated for 2 weeks to form colonies. Then, cells were fixed with 4% paraformaldehyde and stained with 0.1% crystal violet. Visible colonies with more 50 cells were manually counted. The experiments were duplicated in three times.

### Statistical Analysis

All data were analyzed using SPSS 19.0 (SPSS, Chicago, IL, USA). Data were represented as mean ± SD. The grouped t-test was used to compare between-group differences. All statistical tests employed a level of significance of α = 0.05 at 95% confidence level.

## Results

### CircRNA Expression Profile

**Figure 1:**
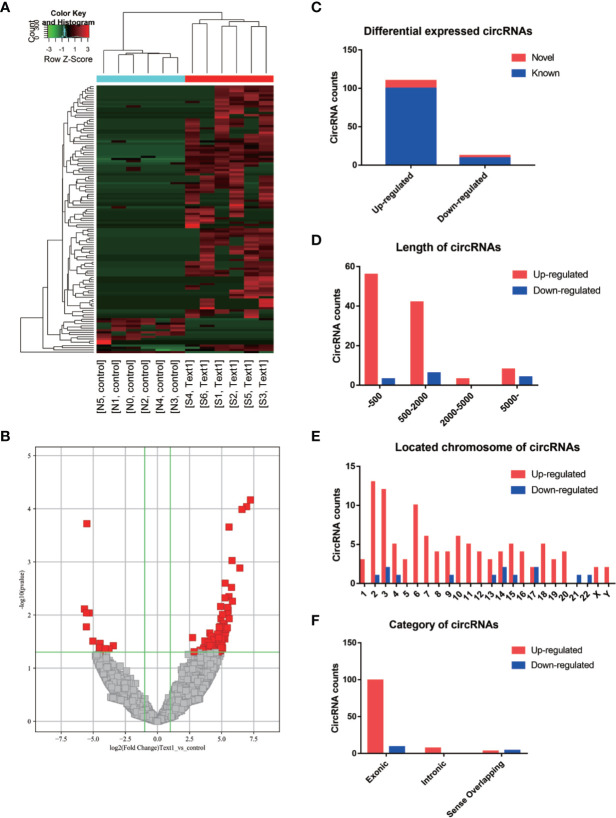
Identification of DE circRNAs in OSCC. **(A)** The characteristics of statistically significantly DE circRNAs with heatmap. **(B)** The characteristics of statistically significantly DE circRNAs with volcano plots. **(C)** The number of statistically significantly DE circRNAs. **(D)** The length of statistically significantly DE circRNAs. **(E)** The chromosomal localization of statistically significantly DE circRNAs. **(F)** The category of statistically significantly DE circRNAs.

We analyzed the circRNA expression profile in 6 pairs of samples of OSCC and normal oral mucosal tissues. A total of 21,505 DE circRNAs were detected, including 16,420 upregulated and 5,085 downregulated circRNAs. One hundred and twenty-two DE circRNAs, among which 109 ones were upregulated and 13 ones were downregulated, fitted the standard (FC ≥ 2 and *P*≥0.05) and were considered statistically significant. The statistically significantly DE circRNAs are shown in a cluster heatmap (**Figure 1A**) and volcano plots (**Figure 1B**).

Among the 122 DE circRNAs, 12 circRNAs were identified for the first time as novel circRNAs. The other 110 circRNAs can be searched in the existing circRNA database (**Figure 1C**). The lengths of most of the 122 DE circRNAs were less than 2,000 nucleotides (**Figure 1D**). The 122 DE circRNAs were localized in all human chromosomes (**Figure 1E**). The circRNAs, based on the sequences, were categorized into 3 types, including exonic circRNAs consisting of only exonic sequences, intronic circRNAs consisting of only intronic sequences, and sense overlapping circRNAs consisting of both exonic and intronic sequences. Among the 122 DE circRNAs, there were 108 exonic circRNAs, 7 intronic circRNAs, and 7 sense-overlapping circRNAs (**Figure 1F**).

### Validation for Top 10 DE CircRNAs

**Figure 2:**
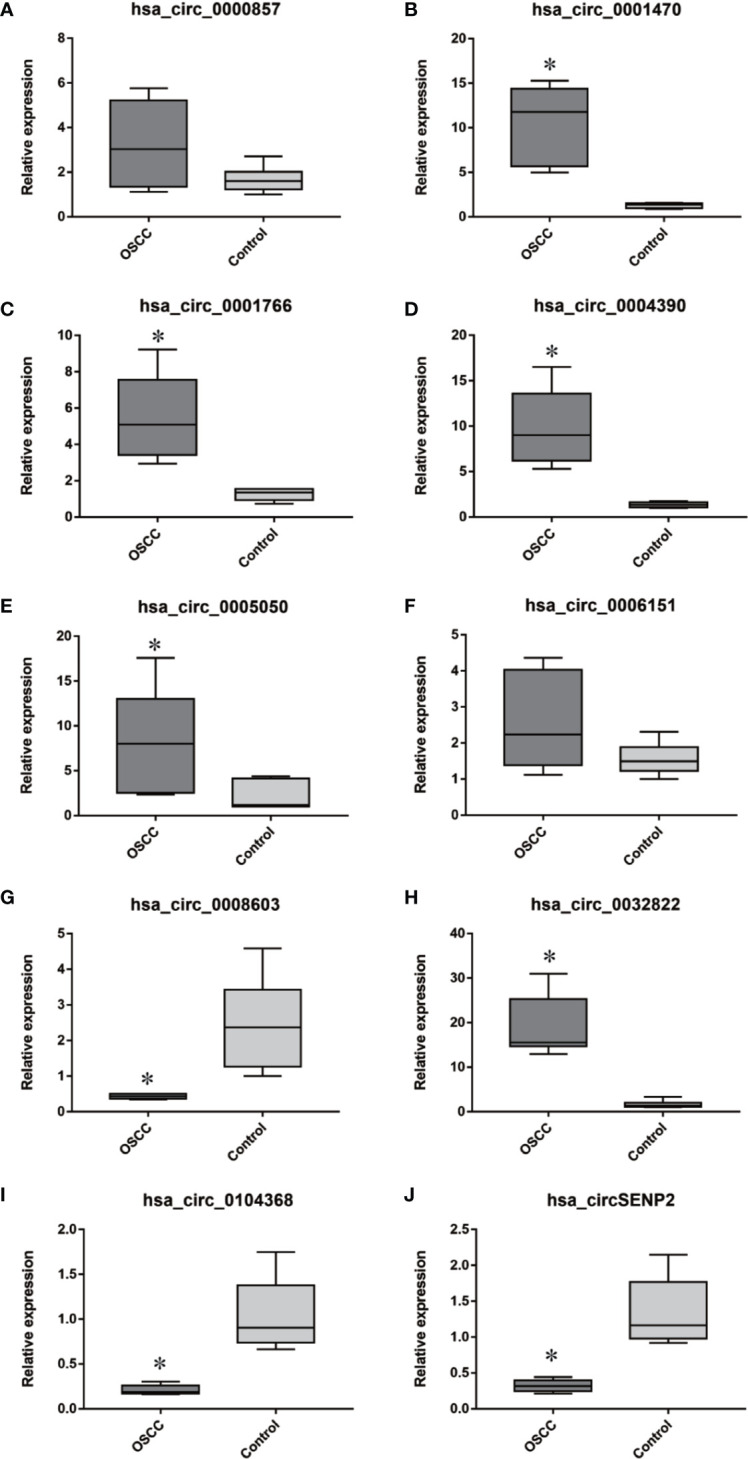
Eight out of 10 circRNAs showed the same directions and statistical significance as the expression changes of the circRNA sequencing, using PCR (*P < 0.05). **(A–J)** Expression of hsa_circ_0000857, hsa_circ_0001470, hsa_circ_0001766, hsa_circ_0004390, hsa_circ_0005050, hsa_circ_0006151, hsa_circ_0008603, hsa_circ_0032822, hsa_circ_0104368, hsa_circSENP2 in OSCC tissues and normal oral mucosal tissues.

According to the fold changes, *P* values, and cancer-association of the DE circRNAs between the 6 pairs of samples of OSCC and normal oral mucosa tissues, we selected the top 10 DE circRNAs (7 upregulated and 3 downregulated) for validation in the same samples from 6 pairs of patients by qRT-PCR. The results suggested that 8 out of 10 circRNAs showed the same trends of expression changes significantly as the circRNA sequencing (**Figure 2**). Then, Sanger sequencing and RNase R assay were done. Two out of 10 circRNAs (hsa_circ_0005050 and hsa_circ_0001766) were identified as DE circRNAs. The biological features of the 2 validated circRNAs are summarized in **Table 1**.

**Table 1:** Biological features of validated circular RNAs (circRNAs).

CircRNA ID	GeneName	CircBase ID	Relative levels	P value	Category	Length
chr2:61712903-61717911-	XPO1	has_circ_0005050	3.921	0.031	Exonic	621
chr7:148716084-148718239-	PDI4	hashsa_circ_0001766	4.373	0.002	Exonic	387

### Bioinformatics Analysis

**Figure 3:**
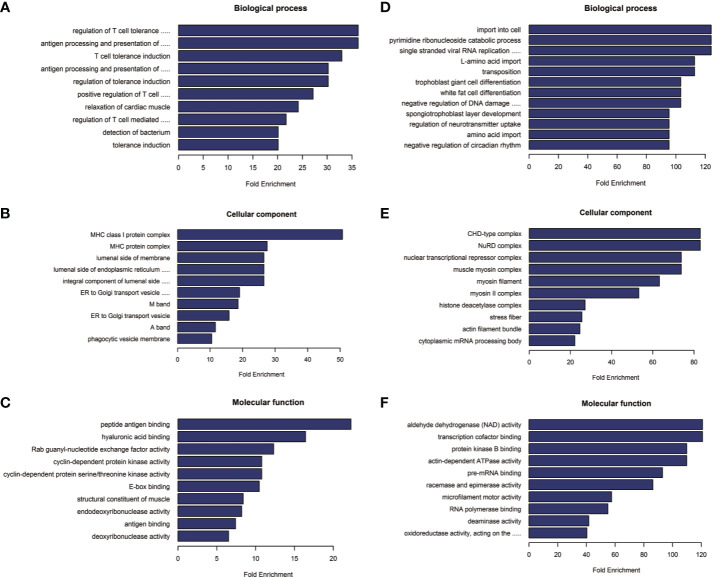
**(A–C)** The top annotation cluster terms of upregulated circRNAs. **(D–F)** The top annotation cluster terms of downregulated circRNAs.

GO and KEGG analyses were used to predict the role of DE circRNAs. We predicted the function of host genes by GO enrichment analysis, based on biological process, cellular components, and molecular function. Top annotation cluster terms (collective biological process, cellular components and molecular function) of upregulated and downregulated circRNAs are shown in **Figure 3**. In addition, possible functions of validated circRNAs are shown in **Table 2**.

**Table 2:** Biological processes of validated circRNAs.

circRNA ID	GO ID	GO terms
hsa_circ_0005050	GO:1901363; GO:0097159; GO:0005488; GO:0003676;	Heterocyclic compound binding; Organic cyclic compound binding; Binding; Nucleic acid binding;
hsa_circ_0001766	GO:1901363; GO:0097159; GO:0005488; GO:0003676;	Heterocyclic compound binding; Organic cyclic compound binding; Binding; Nucleic acid binding;

KEGG pathway analysis was performed to elucidate the role of host genes of circRNAs in different pathways. Thirty pathways that were related to the functions of 30 DE circRNAs were found in the KEGG analysis. In the validated DE circRNAs, hsa_circ_0001470 was involved in transcriptional misregulation in cancer (hsa05202), whereas hsa_circ_0004390 was involved in pathways in cancer (hsa05200) and Rap1 signaling pathway (hsa04015), which have been reported to contribute to the regulation of tumorigenesis and tumor progression (**Figure 4**) (13).

**Figure 4:**
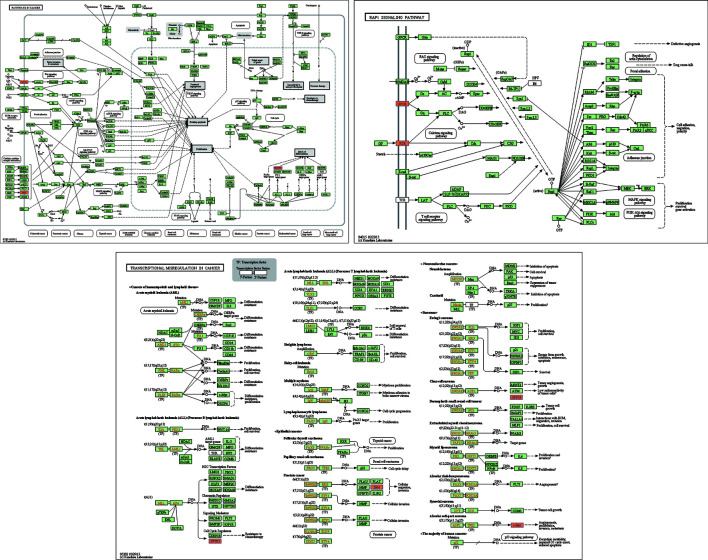
Transcriptional misregulation in cancer (hsa05202), Rap1 signaling pathway (hsa04015), and pathways in cancer (hsa05200).

### Hsa_circ_0001766 Could Potentially Act as a ceRNA in OSCC

Considering the *P* values in the qRT-PCR results, hsa_circ_0001766 was chosen to carry out further research. CircRNAs could regulate the activities of miRNAs by miRNA sponging, thereby regulating gene expression. The binding relationship between hsa_circ_0001766 and miR-877-3p was predicted by TargetScan 7.0 and miRanda v3.3a, which showed that hsa_circ_0001766 might be a ceRNA of miR-877-3p. It has been reported that miR-877-3p targets VEGFA and negatively regulates VEGFA (14). Moreover, multiple studies have demonstrated the role of VEFGA in OSCC (15–18). Hence, we hypothesized that hsa_circ_0001766 sponged miR-877-3p to upregulate VEGFA expression.

**Figure 5:**
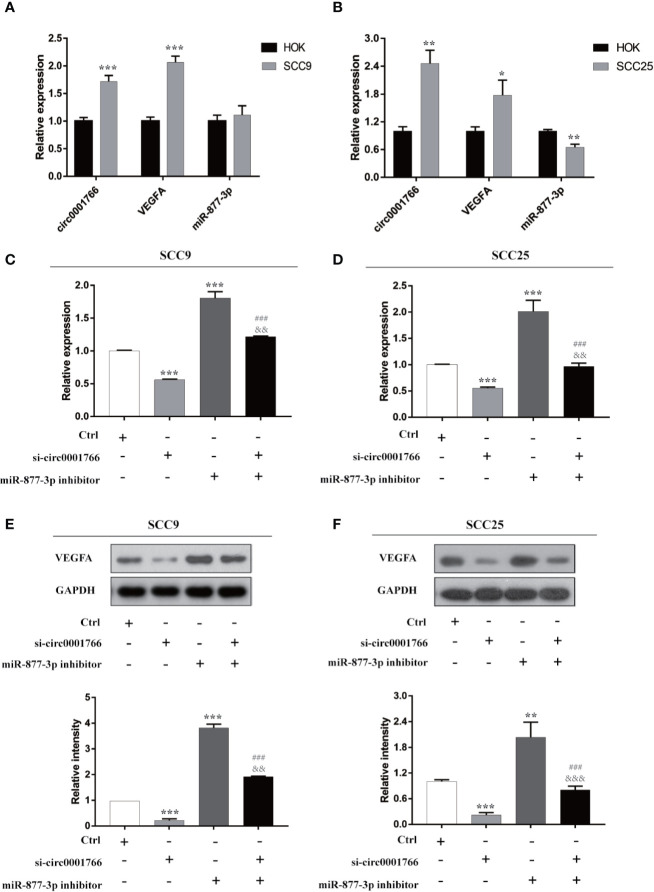
miR-877-3p inhibitor reversed the inhibitory role of si-circ0001766 on expression of VEGFA in SCC9 and SCC25 cell lines. **(A, B)** circ0001766 and VEGFA were upregulated in SCC9 and SCC25. miR-877-3p was downregulated in SCC25. **(C–F)** Identification of the effects of si-circ0001766 and miR-877-3p on VEGFA in SCC9 and SCC25, using PCR and western blotting. * vs Ctrl p < 0.05; ** vs Ctrl p < 0.01; *** vs Ctrl p < 0.001; ^###^ vs si-circ0001766 p < 0.001; ^&&^ vs miR-877-3p inhibitor p < 0.001; ^&&&^ vs miR-877-3p inhibitor p < 0.001.

**Figure 6:**
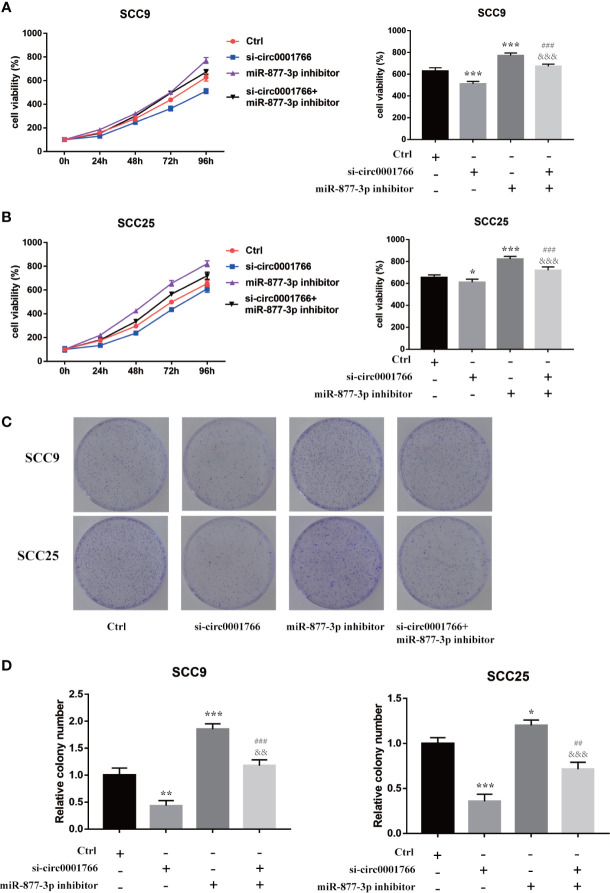
miR-877-3p inhibitor reversed the inhibitory role of si-circ0001766 on expression of cell proliferation in SCC9 and SCC25 cell lines. **(A, B)** CCK assays revealed the effects of circ0001766 and miR-877-3p on cell proliferation in SCC25 and SCC25 cell lines. **(C, D)** Colony formation assay was used to evaluate cell viability after knocking down of circ0001766 and/or miR-877-3p. * vs Ctrl p < 0.05; ** vs Ctrl p < 0.01; *** vs Ctrl p < 0.001; ^##^ vs si-circ0001766 p < 0.01; ^###^ vs si-circ0001766 p < 0.001; ^&&^ vs miR-877-3p inhibitor p < 0.001; ^&&&^ vs miR-877-3p inhibitor p < 0.001.

Subsequently, we investigated the possible role of hsa_circ_0001766/miR-877-3p/VEGFA in OSCC. Compared with HOK cells, in OSCC cell lines SCC9 and SCC25, expressions of hsa_circ_0001766 and VEGFA mRNA were significantly upregulated (**Figures 5A, B****)**. Using qRT-PCR and western blotting, we identified the role and effect of hsa_circ_0001766 and miR-877-3p on VEGFA. While knockdown of hsa_circ_0001766 inhibited the expression of VEGFA, miR-877-3p inhibitor reversed this inhibitory effect of si-hsa_circ_0001766 on expression of VEGFA (**Figures 5C–F**). To elucidate the role of hsa_circ_0001766 on OSCC cell proliferation, CCK8 and colony formation assays were done (**Figure 6**). In SCC9 and SCC25 cell lines, knockdown of hsa_circ_0001766 suppressed cell proliferation. However, when SCC9 and SCC25 cells were treated with si-hsa_circ_0001766 plus miR-877-3p, the inhibitory effect of si-circ001766 on cell proliferation was inversed by underexpression of miR-877-3p. These findings indicated that hsa_circ_0001766 might act as a ceRNA in OSCC and play an important role in OSCC cell proliferation through the hsa_circ_0001766/miR-877-3p/VEGFA axis.

## Discussion

CircRNA is a new class of RNA, which is widely expressed in many animals. Since the publications on circRNAs by Memczak and Hansen in 2013 (3, 4), an increasing number of studies have found that circRNAs are involved in the pathogenesis of various human diseases. The functions of circRNA include protein binding, miRNA sponging, and promoting host gene expression and protein translation. In recent years, circRNAs have been shown to play an important role in the pathogenesis of cancer and could be potential biomarkers for this disease.

In this study, we investigated the circRNA expression profiles in 6 pairs of samples of OSCC and normal oral mucosal tissues. One hundred and twenty-two DE circRNAs, among which 109 were upregulated and 13 were downregulated, were considered statistically significant. We validated 8 circRNAs by qRT-PCR, which showed the same directions and statistical significance as the expression changes of the circRNA sequencing. Then, hsa_circ_0005050 and hsa_circ_0001766 were identified as DE circRNAs using Sanger sequencing and RNase R assay. Hsa_circ_0005050 is spliced from XPO1, which could regulate the nuclear export of cellular proteins. Hsa_circ_0005050 has been reported to be differential expressed in many cells and tissues, including platelets, brain, epidermal keratinocytes, and liver cancer cells (19–21). Hsa_circ_0001766 is derived from PDIA4, which encodes protein disulfide isomerase in the endoplasmic reticulum. To our knowledge, this is the first study to report that hsa_circ_0001766 is upregulated in OSCC. After taking the *P* values in the results of qRT-PCR into consideration, hsa_circ_0001766 was selected to explore further the role of circRNA in OSCC.

The results of GO enrichment analysis suggest that DE circRNAs participate in numerous biological processes. The biological processes that upregulated circRNAs participate in include T cell tolerance induction, regulation of T cell tolerance induction, positive regulation of T cell-mediated cytotoxicity, and regulation of T cell-mediated immunity. Sun et al. (22) found that regulatory T cells are important components in the tumor microenvironment and play a role in local immune regulation. CD4+ T cells increased gradually with the progression of oral dysplasia in oral mucosa. This suggested that the local change to immunosuppressive cells had significant effects on progression of OSCC (22). Furthermore, it is reported that Gal3, that is highly expressed in OSCC, on one hand, is involved in negative selection of T cells during thymic T cell maturation and, thereby promotes central immune tolerance, and on the other hand, Gal3 can act as an immune checkpoint to inhibit T cell activation and thus promotes peripheral immune tolerance. Immune tolerance plays an important role in progression of OSCC (23). The biological processes that downregulated circRNAs participate in include regulation of neurotransmitter uptake and negative regulation of the circadian rhythm. The body clock has an effect on cells and tissues through endocrine and neurotransmitter regulation; the circadian rhythm plays an important role in the development of tumors, and in the therapeutic and side effects of anticancer drugs (24). These indicate that DE circRNAs may modulate the aforementioned biological processes to influence OSCC progression. The results of KEGG analysis suggested that hsa_circ_0001470 is involved in transcriptional deregulation in cancer, whereas hsa_circ_0004390 is involved in pathways in cancer and Rap1 signaling pathway, which have been reported to contribute to the regulation of tumorigenesis and tumor progression (13). This may perhaps explain the role of circRNAs in the pathogenesis of OSCC.

CircRNAs can regulate the activities of miRNAs by miRNA sponging and, thereby participate in the pathogenesis of cancer. In our study, binding relationships between circRNAs and miRNAs were predicted by TargetScan 7.0 and miRanda v3.3a. The results of prediction showed that hsa_circ_0001766 might act as a ceRNA and targets miR-877-3p. It has been reported that miR-877-3p targets VEGFA and negatively regulates VEGFA (14). Positive expression of VEGFA was associated with significantly poor prognosis in OSCC (15, 16). VEGFA contributed to OSCC cell proliferation and tumor initiation (17, 18). In OSCC cell lines SSC9 and SCC25, hsa_circ_0001766 and VEGFA were upregulated. Subsequently, miR-877-3p inhibitor reversed the inhibitory effect of si-hsa_circ_0001766 on inhibition of VEGFA. We further investigated the role of hsa_circ_0001766 in OSCC cell proliferation. While knockdown of hsa_circ_0001766 suppressed cell proliferation, miR-877-3p inhibitor reversed the inhibitory effect of si-hsa_circ_0001766 on cell proliferation. These findings indicated hsa_circ_0001766 might act as a ceRNA in OSCC and played an important role in OSCC cell proliferation through the hsa_circ_0001766/miR-877-3p/VEGFA axis.

In conclusion, we have established that numerous circRNAs were dysregulated in OSCC and normal oral mucosal tissues in this study. Results of bioinformatics analysis implied that circRNAs were enriched in many cancer-related biological processes and might be involved in these pathways. However, this study has a few limits, such as limited numbers of patients and using normal tissues in the non-cancer patients as control. Then, we identified the potential role of the hsa_circ_0001766/miR-877-3p/VEGFA axis in OSCC cell proliferation. Our study not only reveals the possible role of circRNAs in the progression of OSCC, but also provides a base for future research on elucidation of new biomarkers in OSCC.

## Data Availability Statement

The datasets generated for this study can be found in the GENE EXPRESSION OMNIBUS GSE131182.

## Ethics Statement

The studies involving human participants were reviewed and approved by The Ethics Committee of Shanghai Ninth People’s Hospital. The patients/participants provided their written informed consent to participate in this study.

## Author Contributions

HZ and SL conceived the study. YXS, YHS, and SX conducted the experiments, analyzed the data, and wrote the manuscript. All authors contributed to the article and approved the submitted version.

## Funding

This research was supported by the National Key R&D Program of China (2017YFC0840100, 2017YFC0840110), National Construction Project of Clinical Key Specialized Department (2013) 544, Shanghai Science and Technology Commission (Grant No. 20151005), and Shanghai Health Development Planning Commission (Grant No. 201540257). We would like to appreciate all the staff of the Department of Oral Mucosal Diseases for supporting this study.

## Conflict of Interest

The authors declare that the research was conducted in the absence of any or financial relationships that could be construed as a potential conflict of interest.
